# Transfer of stabilising mutations between different secondary active transporter families

**DOI:** 10.1002/2211-5463.13168

**Published:** 2021-05-08

**Authors:** Cristina Cecchetti, Nicola J. Scull, Thotegowdanapalya C. Mohan, Yilmaz Alguel, Alexandra M. C. Jones, Alexander D. Cameron, Bernadette Byrne

**Affiliations:** ^1^ Department of Life Sciences Imperial College London UK; ^2^ School of Life Sciences University of Warwick Coventry UK; ^3^ Present address: Department of Biotechnology and Bioinformatics Faculty of Life Sciences JSS Academy of Higher Education and Research Mysore Karnataka 570015 India

**Keywords:** integral membrane protein, mutagenesis, secondary active transporter, stability, structural studies

## Abstract

Integral membrane transporters play essential roles in the movement of substrates across biological membranes. One approach to produce transporters suitable for structural studies is to introduce mutations that reduce conformational flexibility and increase stability. However, it can be difficult to predict which mutations will result in a more stable protein. Previously, we stabilised the uric acid‐xanthine transporter, UapA, a member of the SLC23 family, through introduction of a single‐point mutation, G411V, trapping the protein in the inward‐facing conformation. Here, we attempted to stabilise the structurally related BOR1 transporter from *Arabidopsis thaliana*, a member of the SLC4 family, by introducing the equivalent substitution. We identified possible residues, P362 and M363, in AtBOR1, likely to be equivalent to the G411 of UapA, and generated four mutants, P362V or L and M363F or Y. Stability analysis using heated Fluorescent Size Exclusion Chromatography indicated that the M363F/Y mutants were more stable than the WT AtBOR1 and P362V/L mutants. Furthermore, functional complementation analysis revealed that the M363F/Y mutants exhibited reduced transport activity compared to the P362V/L and WT proteins. Purification and crystallisation of the M363F/Y proteins yielded crystals that diffracted better than WT (5.5 vs 7 Å). We hypothesise that the increased bulk of the F and Y substitutions limits the ability of the protein to undergo the conformational rearrangements associated with transport. These proteins represent a basis for future studies on AtBOR1.

## Graphical abstract

We transferred a stabilising substitution (G411V) in the membrane transporter UapA from *Aspergillus* *nidulans* to the structurally related BOR1 transporter from the model plant, *Arabidopsis thaliana*. Amino acid substitutions M363F and M363Y result in increased stability of AtBOR1 associated with reduced transport activity and thus reduced conformational flexibility. AtBOR1 M363 is likely equivalent to UapA G411.

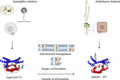

**Abbreviations**
AtBOR1: *Arabidopsis thaliana* boron transporter 1
DDM: dodecyl β‐D‐maltoside
DM: decyl β‐D‐maltoside
FSEC: fluorescent size exclusion chromatography
hFSEC: heated fluorescent size exclusion chromatography
hAE1: human Anion Exchanger 1 protein
NBCe1: human sodium‐coupled acid–base transporter
ScBOR1p: *Saccharomyces cerevisiae* boron transporter
SLC: solute carrier
TEV: tobacco etch virus
TM: transmembrane domain
UapA: uric acid‐xanthine transporter
WT: wild‐type
yEGFP: yeast enhanced green fluorescent protein

Secondary active transporters are essential mediators of cellular uptake and export. They transport a wide range of substrates which bind to substrate‐specific binding sites on one side of the membrane. Upon binding of both the substrate and the cotransported ion, the transporter undergoes a series of major conformational rearrangements to both close access on the substrate‐binding side of the membrane and open access on the opposite side of the membrane [1]. Understanding of precisely how transporters perform their function has greatly increased in the last decade due to a substantial number of high‐resolution crystallographic structures [2]. However, we still lack a detailed understanding of the mechanism of many transporters, particularly those from eukaryotic sources. A key difficulty associated with structural studies of integral membrane transporters relates to their conformationally dynamic nature, associated with a lack of stability particularly in the detergent‐based solutions required for membrane extraction, isolation and crystallisation. Researchers have employed a number of approaches to limit protein flexibility and trap transporters into single conformational states, including the use of inhibitors [3], antibodies or nanobodies [4] and introduction of mutations [5, 6]. Often combinations of these approaches are used; Fab fragment and mutations [7], mutations and an alternative substrate [8, 9], mutagenesis and a chimera of two transporters [10].

Our group solved the structure of the H^+^‐dependent uric acid‐xanthine transporter, UapA from the model organism, *Aspergillus nidulans* [11] in the inward‐facing conformation. In the case of UapA, the wild‐type (WT) form of the protein expressed well and could be isolated to high homogeneity [12]. However, the isolated WT protein was relatively unstable, undergoing degradation at both 20 and 4 °C [13]. Based on previous studies on UapA, six single‐point mutations were identified which retained the ability to traffic to the membrane in *A. nidulans* but were no longer transport active [14, 15]. These features indicated that the mutants were correctly folded but might be conformationally restricted. Screening of these mutants identified one, G411V, as exhibiting much greater stability than the WT protein at elevated temperature, in a relatively harsh detergent and during long‐term storage at both 20 and 4 °C [13]. It was this mutant that yielded the high‐resolution structure of the inward‐facing conformation of UapA, although it should be noted that an additional truncation of the first 11 amino acids was required to yield well‐diffracting crystals.

UapA seems to function via an elevator mechanism [2], whereby the core or substrate‐binding domain [made up of transmembrane domain (TMs) 1, 2, 3, 4, 8, 9, 10 and 11] undergoes a translation and rotation motion through the membrane, moving against the gate or dimerisation domain (made up of TMs 5, 6, 7, 12, 13 and 14). The Val side chain in the G411V mutant projects into the translocation channel, and it is possible that the additional bulk of this residue, compared with the WT Gly residue, prevents this movement of the core domain from taking place, locking the protein in the inward‐facing conformation.

UapA belongs to the SLC23 family of transporters and is a structural homologue of the SLC4 and SLC26 transporters, all of which are suggested to also function via an elevator mechanism. Several of these have been structurally characterised including the human Anion Exchanger 1 protein [16], the human sodium‐coupled acid–base transporter, NBCe1 [17], the borate transporter from *Arabidopsis thaliana*, AtBOR1 [18], the cyanobacteria bicarbonate transporter [19] and the prokaryotic fumarate transporter SLC26Dg [20]. Some of these were artificially stabilised; for example, the hAE1 and the SLC26Dg were crystallised as complexes with antibody fragments or nanobodies. However, it is unclear whether it is possible to stabilise proteins from the SLC4 and SLC26 families using the equivalent residue to G411V. A structure of a truncated version of AtBOR1 is available but only at low resolution and lacking almost all the loop regions. Thus, we attempted to stabilise the AtBOR1 by introducing mutations at positions Pro362 and Met363, both near to the position of G411 of UapA. Our analysis indicated that mutations at M363 had stabilising effects on the protein, with M363F inducing the greatest stability, and demonstrated that stabilising mutations can be transferred between structurally related proteins.

## Materials and methods

### Mutagenesis

All the mutants were generated using the QuikChange Lightning Site‐Directed Mutagenesis Kit (Agilent) using the primers (Table S1) and the AtBOR1 WT gene as template.

### Protein expression and membrane preparation

The WT AtBOR1 and the different mutants were individually expressed as fusions with a C‐terminal tobacco etch virus (TEV)‐cleavable yEGFP His‐tag in the pDDGFP‐2 plasmid [21]. All the proteins were expressed as previously described [21, 22] in FGY217 *Saccharomyces cerevisiae* cells. In brief, individual colonies were inoculated in 50 mL of ‐URA media (2 g·L^−1^ amino acid mix w/o uracil, 6.7 g·L^−1^ yeast nitrogen base w/o amino acids) supplemented with 0.01% w/v glucose. The cells were cultured to an OD_600_ = 0.6 at 30 °C and protein expression induced by the addition of 2% galactose. The culture was incubated at 30 °C for 22 h with 300 r.p.m. shaking. The cells were harvested by centrifugation at 4000 ***g*** for 10 min at 4 °C and then resuspended in the membrane resuspension buffer (50 mm Tris/HCl pH 7.5, 1 mm EDTA and 0.6 m sorbitol supplemented with 1× cOmplete EDTA‐free protease inhibitor cocktail tablet (Roche, Basel, Switzerland). The expression level was assessed by measuring the fluorescence signal of 200 µL cells in membrane resuspension buffer using a SpectraMax M2e (Molecular Devices, San Jose, CA, USA), with an excitation wavelength of 488 nm and emission wavelength of 512 nm. Following the addition of 500 µL of 0.5 mm glass beads, the cells were lysed using a Biomedical MMP FastPrep‐24 5G benchtop cell homogeniser with six cycles of 20 s each followed by a 2‐min incubation at 4 °C. The cell lysates were submitted to differential centrifugation at 14 000 ***g*** for 30 s followed by 22 000 ***g*** for 1 h at 4 °C. The resultant membrane pellets were resuspended in 900 µL of 20 mm Tris/HCl pH 7.5 and 0.3 m sucrose. For large scale expression, protein was produced in a total volume of 6 L of ‐URA media culture and the cells lysed using a cell disruptor (Constant Systems, Daventry, Northants, UK) at 25, 30, 33 and 36 kpsi at 4 °C. Any unbroken cells were separated by centrifugation at 10 000 ***g*** for 10 min at 4 °C, with the membranes harvested by ultracentrifugation at 100 000 ***g*** for 2 h at 4 °C. The membranes were resuspended in 6 mL of 20 mm Tris/HCl pH 7.5 and 0.3 m sucrose per litre of cell culture and either used immediately or snap‐frozen in liquid nitrogen and stored at −80 °C until further use.

### FSEC and hFSEC

Membranes containing the individual AtBOR1 constructs were diluted in solubilisation buffer (1× PBS pH 7.5, 1% w/v dodecyl β‐D‐maltoside (DDM) supplemented with 1 tablet of protease inhibitor) to a final protein concentration of ~ 50 µg·mL^−1^. The samples were incubated for 1 h with gentle mixing at 4 °C, followed by centrifugation at 14 000 ***g*** for 1 h at 4 °C to remove insoluble material. A 500 µL aliquot of the soluble supernatant was injected onto a Superose 6 10/300 column equilibrated with 20 mm Tris/HCl (pH 7.5), 150 mm NaCl and 0.03% DDM and the fractions collected in 200 µL volume in a clear‐bottomed black 96‐well microplate. The GFP fluorescence of the fractions was measured using a SpectraMax M2e (Molecular Devices) fluorimeter, with an excitation wavelength of 488 nm and emission wavelength of 512 nm. For heated FSEC (hFSEC) experiments, the supernatant was incubated at 46 °C for 10 min, centrifuged at 14 000 ***g*** for 10 min and then loaded onto a Superose 6 10/300 column and repeating the procedure as described above.

### Localisation

Yeast cultures expressing the AtBOR1 constructs were centrifuged briefly to harvest the cells which were then resuspended in 100 µL of 4% paraformaldehyde. After a 15‐min incubation at room temperature, the cells were washed twice with 1 mL and then 20 µL of 1.2 m sorbitol, 16.6 mm KH_2_PO_4_ and 83.4 mm K_2_HPO_4_. A 3–5 µL aliquot of the fixed cells containing the different AtBOR1 constructs was individually dispensed onto a microscope cover slide and imaged on a Zeiss (Oberkochen, Germany) LSM‐520‐inverted confocal microscope using a 63× oil immersion lens.

### Functional complementation assay

The different AtBOR1 constructs were individually transformed into the FGY217 ∆*bor1p* knockout *S. cerevisiae* strain [23]. Transformants were inoculated in 10 mL of ‐URA media supplemented with 2% w/v glucose and incubated overnight at 30 °C, 300 r.p.m. After a fivefold serial dilution with ‐URA media of the initial culture at OD_600_ = 0.5, 10 µL of each dilution was spotted on ‐URA plates containing 2% galactose supplemented with 0, 5 and 7.5 mm boric acid. The plates were then incubated at 30 °C for 7 days and imaged with a ChemiDoc MP Imaging System (Bio‐Rad, Hercules, CA, USA).

### Isolation of mutant proteins

Membranes containing the mutant proteins, from a 6 L expression volume, were solubilised for 1 h at 4 °C in solubilisation buffer (1×PBS pH 7.4, 100 mm NaCl, 10% v/v glycerol, 1% w/v DDM) with a cOmplete EDTA‐free protease inhibitor cocktail tablet (Roche). The insoluble membranes were pelleted at 100 000 ***g*** for 45 min at 4 °C, and the supernatant, supplemented with 10 mm imidazole, was loaded onto 5 mL His‐Trap pre‐equilibrated with 1×PBS (pH7.4), 100 mm NaCl, 10% w/v glycerol and 0.03% v/v DDM. After washing with increasing concentrations of imidazole (20, 40, 60 mm), the protein was eluted with 1xPBS (pH 7.4), 100 mm NaCl, 10% w/v glycerol, 0.03% v/v DDM and 350 mm imidazole. The protein concentration in the eluate was estimated via GFP fluorescence measurement [21] and the sample was incubated (1 : 10 dilution) in 20 mm Tris/HCl (pH 7.5), 150 mm NaCl, 0.03% w/v DDM for 16 h at 4 °C with His‐tagged TEV protease (1 : 1 ratio). After a reverse‐IMAC step in the His‐Trap, the protein was eluted from a Superdex 200 10/300 column in [20 mm Tris/HCl (pH 7.5), 150 mm NaCl and 0.03% w/v DDM. The quality of the protein was assessed on a 12%Tris‐Glycine SDS/PAGE gel, and the central fractions were pooled together and concentrated to 10 mg·mL^−1^ for crystallisation trials.

### Crystallisation and crystal harvesting

AtBOR1 M363F and AtBOR1 M363Y purified in the presence of 3 x CMC DDM or decyl β‐D‐maltoside (DM). A variety of commercial screens (MemGold, MemGold2, MemTrans; Molecular Dimensions, Sheffield, UK) were employed to set up preliminary crystallisation trials by the Mosquito crystallisation robot (SPT LabTech, Melbourn, Herfordshire, UK) in sitting drop vapour diffusion (100 nL protein + 100 nL crystallisation condition and 85 µL reservoir). AtBOR1 M363F in DM and DDM was also used to set up crystallisation trials by hand (1 µL protein and 1 µL condition, 850 µL reservoir) for optimisation of some conditions identified from the commercial screens. Additive and detergent screens (Hampton Research, Aliso Viejo, CA, USA) were also used with the following settings: 20 nL additive/detergent in the drop made of 100 nL protein and 100 nL crystallisation condition (9% PEG_8000_, 0.1 m MOPS pH 7), 85 µL reservoir. All buffers were titrated with NaOH. The plates were stored at 4 and 20 °C. Crystals were shipped to Diamond Light Source, Oxfordshire, UK, and screened on either i04 or the Microfocus beamline i24.

## Results

### P362 and M363 of AtBOR1 are possible equivalent residues to G411 of UapA

**Fig. 1:**
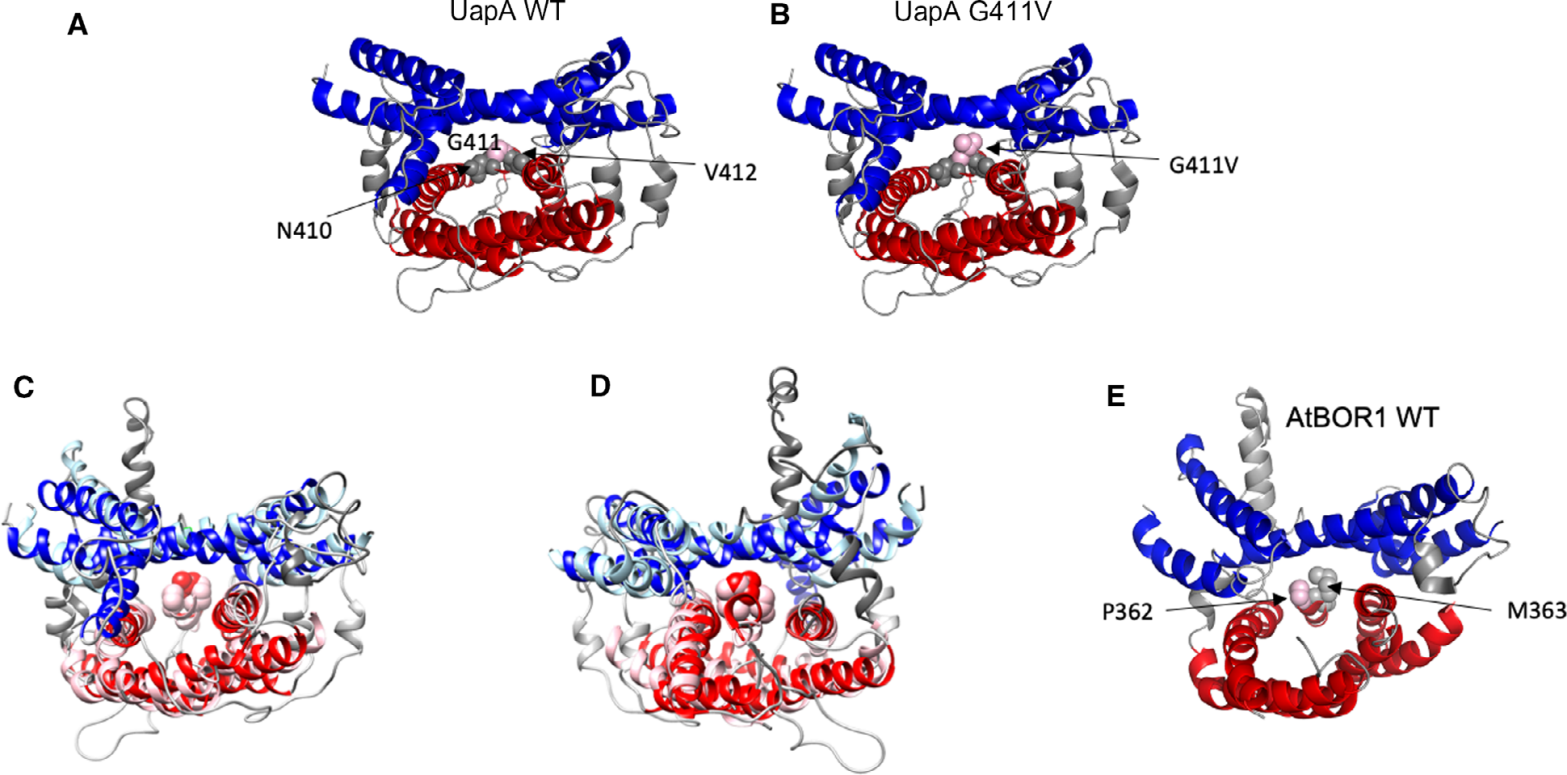
Identifying the equivalent residues to UapA G411V in AtBOR1 (A) Structure of UapA (PDB: 5I6C) in the inward‐facing conformation with the WT Gly residue at position 411. The core domain helices are coloured red, the gate domain helices are coloured blue with interconnecting regions shown in grey. G411 is indicated in space‐filling representation in pale pink, and the flanking residues, N410 and V412 are shown in space‐filling representation in grey. (B) Structure as in (A) but with the G411V substitution as obtained in the actual structure of the protein. (C, D) Alignment of UapA and AtBOR1. The gate and core domains are coloured blue (UapA) and light blue (AtBOR1), and the core domains are coloured red (UapA) and light pink (AtBOR1). V411 seen in the structure of UapA and possible structurally equivalent residues P362 and M363 of AtBOR1 are shown in space‐filling representation. C and D show the proteins from the extracellular and intracellular sides of the membrane, respectively. (E) Structure of the AtBOR1 in the inward occluded state (PDB 5L25), with P362 and M363 shown in space‐filling representation in pale pink and grey, respectively. The core domain helices are coloured red and the gate domain helices are coloured blue with interconnecting regions shown in grey. In all structure images, only a single monomer is represented and TM3 and sections of the loop connecting TM3 and TM4 have been removed for clarity.

A comparison of the UapA WT form (G411) with the mutant that was structurally characterised (G411V) illustrates the difference in bulk introduced by the Val substitution at the interface between the gate (blue) and core (red) domains (Fig. 1A,B). The structures of AtBOR1 (PDB 5L25) and UapA‐G411V_Δ1‐11_ (PDB 56IC) were superposed using UCSF Chimera [24]. UapA‐G411V_Δ1‐11_ is in the inward‐facing conformation while AtBOR1 is suggested to be in the inward occluded conformation. The whole structures superpose with an RMSD of 13.8 Å across all atom pairs (Fig. 1C,D). This superposition suggested that either P362 or M363 of AtBOR1 (Fig. 1E) was the structurally equivalent residue to G411 of UapA. It was difficult to be completely clear given the differences in conformation of the two proteins. We chose to generate P362V and P362L mutants with a view to introducing a side chain that was bulkier than in the WT protein. Given that M363 is already quite a bulky residue, we substituted this with either a Phe or a Tyr.

### AtBOR1 mutants express and traffic similarly to WT

All the mutations expressed to a similar average level as WT (typically ~ 3–4 mg·L^−1^) as assessed by GFP fluorescence measurements (Fig. S1A). Both the WT and mutant forms of the protein traffic to the membrane as assessed by confocal microscopy (Fig. S1B).

### AtBOR1 M363 mutants exhibit enhanced stability compared with WT

**Fig. 2:**
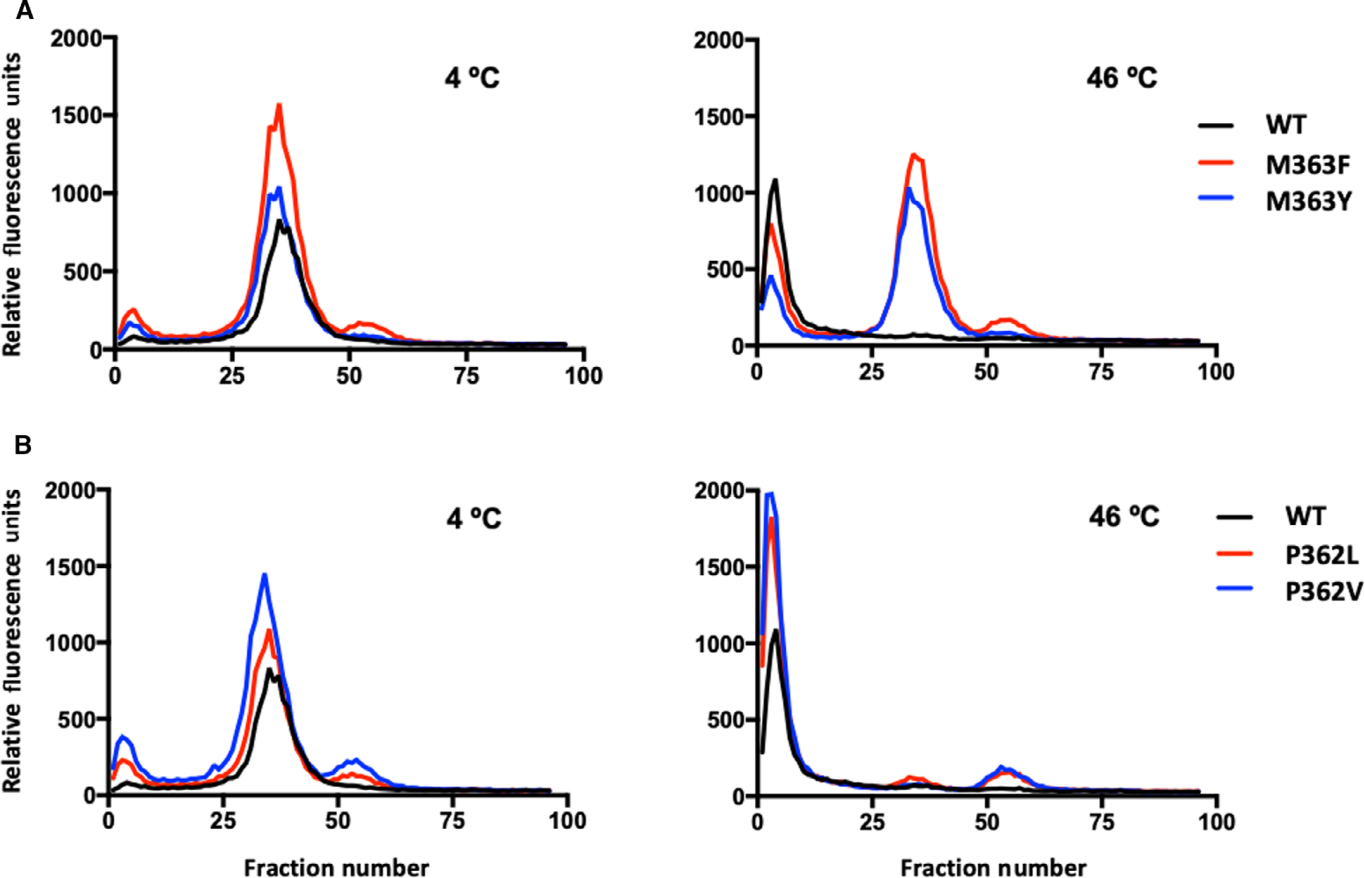
M363 mutants are more stable than WT The mutants and WT AtBOR1 were solubilised in 1% DDM and submitted directly to FSEC (4 °C) or solubilised in 1% DDM and then heated at 46 °C for 10 min prior to centrifugation and column loading. The fluorescence for each fraction was measured and the data normalised based on the maximum monodispersed peak (elutes ~ fraction 35) height obtained for the WT. (A) The FSEC profiles obtained at 4 °C for the M363 mutants and WT or (B) the P362 mutants and WT (left panels) and following heating at 46 °C (right panels). Data shown are representative of 3 independent experiments.

FSEC and hFSEC analysis were used to obtain a T_m_ for AtBOR1 solubilised in 1% DDM of 43 °C (Fig. S2). This was then used as the basis for thermostability analysis of the AtBOR1 mutants. All the AtBOR1 constructs were expressed, solubilised in buffer containing 1% DDM and then submitted to both FSEC and hFSEC analysis. The solubilisation efficiencies were between 62 and 75% (Fig. S1C). All proteins exhibited similar FSEC profiles to the WT (Fig. 2A,B, left panels) with a good recovery of monodisperse soluble protein. Following heating at the increased temperature of 46 °C, there is a substantial loss of monodisperse WT protein; however, both the M363F and M363Y mutants exhibit at least 50% monodisperse protein. In all cases, at this higher temperature there is a substantial increase in the aggregated protein, as shown by the first peak to be observed in the SEC profile, the aggregation peak (Fig. 2A,B). Analysis of the P362 mutants indicates that these are no more stable than WT AtBOR1. The data clearly indicate that the M363F and M363Y constructs are more stable than both the P362 mutants and the WT protein.

### AtBOR1 M363 mutants show dramatically reduced borate efflux activity

**Fig. 3:**
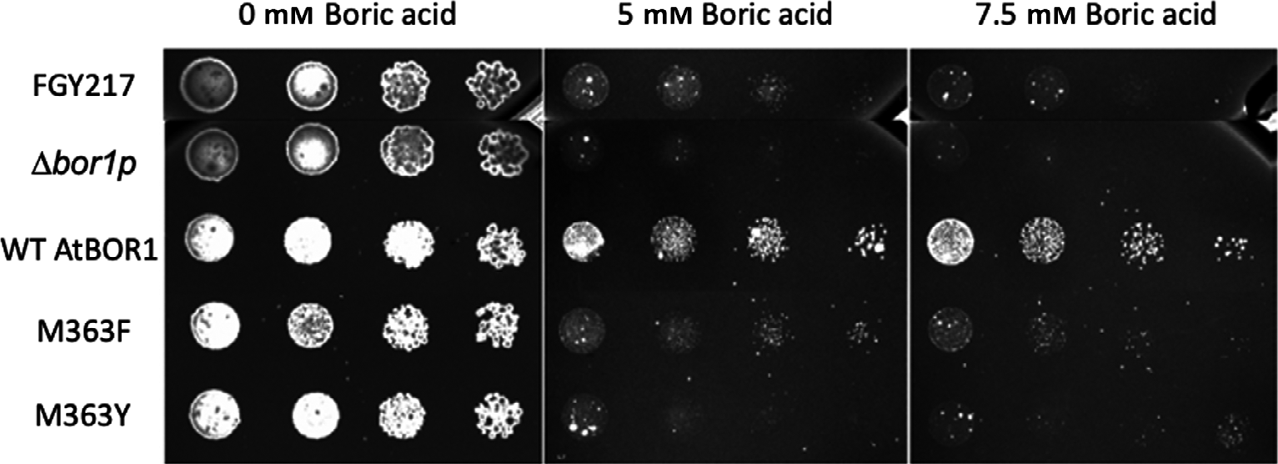
The M363 mutants exhibit reduced boron efflux activity. *S. cerevisiae* cells were spotted in a 5× series of dilution from left to right on ‐URA media supplemented with 2% galactose and 0, 5, or 7.5 mm boric acid. Plates were incubated at 30 °C for 5 days before being imaged. AtBOR1 M363F and M363Y were transformed in the *S. cerevisiae* knockout Δ*bor1p* strain in order to assess the function of these variants. The Δ*bor1p* cells were also transformed with an empty vector as a negative control (Δ*bor1p* in the figure) and FGY217 cells expressing the endogenous ScBOR1p were used to demonstrate endogenous borate efflux (FGY217 in the figure). The Δ*bor1p* cells overexpressing AtBOR1 WT was also analysed as a positive control (WT in the figure).

In order to assess the ability of the mutants to transport borate, we used a *S*. *cerevisiae* Δ*bor1p* knockout strain which lacks the endogenous ScBOR1p. ScBOR1p is responsible for efflux of borate from the yeast cells. Without this protein, intracellular boron builds up to toxic levels and inhibits yeast cell growth. We individually transformed the WT AtBOR1 and different mutants into the Δ*bor1p* strain and assessed cell growth on solid media in the presence of different concentrations of boric acid. The Δ*bor1p* strain (Fig. 3) exhibits almost undetectable levels of growth at 5 and 7.5 mm boric acid. WT AtBOR1 functionally complements the knocked out ScBOR1p allowing effective growth of the yeast cells at even 7.5 mm boric acid (Fig. 3). The cells expressing the WT AtBOR1 show better growth than our standard *S. cerevisiae* expression strain, FGY217, which endogenously expresses ScBOR1p. This is probably the result of higher recombinant expression of the AtBOR1 compared with the native expression levels of ScBOR1p. The P362V/L mutants also effectively complement the loss of ScBOR1p, resulting in yeast cell growth at all boric acid concentrations tested and indicating that these substitutions cause no or little loss in AtBOR1 function (Fig. S3). However, the cells transformed with M363F and M363Y mutants exhibit markedly reduced growth compared with the WT AtBOR1 (Fig. 3). There appears to be some low‐level borate efflux activity retained in these mutants as the cells expressing these proteins did exhibit slightly better growth than the Δ*bor1p* strain at 5 and 7.5 mm boric acid (Fig. 3). These data suggest that the M363F and M363Y mutants function less effectively than either the WT AtBOR1 or the P362 mutants. Given that the M363F/Y mutants appear more stable than the WT protein and exhibit substantially reduced transport activity, it is possible that these variants may be preferentially in a single conformation with very limited capacity to undergo conformational rearrangements associated with substrate transport.

### M363F and M363Y yield more homogenous protein and better diffracting crystals

**Fig. 4:**
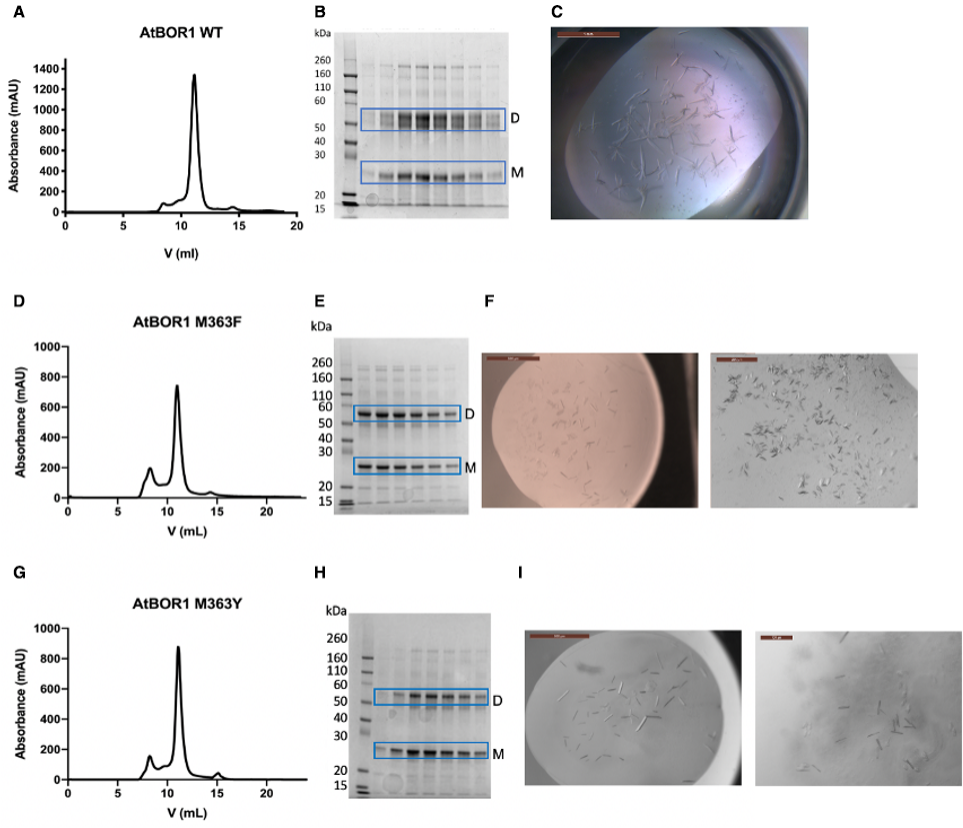
Purification and crystallisation of AtBOR1 M363F and M363Y. Size exclusion chromatography profiles for WT (A), M363F (D) and M363Y (G); V = retention volume (mL). SDS/PAGE analysis of the SEC elution fractions for WT (B), M363F (E) and M363Y (H). The bands corresponding to monomeric (M) and dimeric protein (D) are indicated; kDa = molecular weight markers. (C) shows the WT crystals in 0.1 m Tris pH 7.5, 0.1 m NaCl, 11% PEG_4000_, drop size 2 µL, scale bar = 1 mm. (F) Shows the crystals obtained for M363F purified in DDM and grown in 9% PEG_8000_, 0.1 m MOPS pH 7, 30% w/v D‐(+)‐Glucose monohydrate, drop size 200 nL, scale bar = 500 µm (left), and M363F in DM in 0.2 m Li sulphate, 0.1 m Na chloride, 0.1 m HEPES pH7, 31% v/v PEG_400_ (condition A10 MemGold2), drop size = 200 nL, scale bar = 200 µm (right). (I) Crystals of M363Y in DDM in 0.2 m ammonium acetate, 0.1 m sodium chloride, 0.1 m Bis‐Tris pH 6.3, 12% w/v PEG_4000_ (E1 MemTrans), drop size 200 nL, s Scale bar = 500 µm (left), and of M363Y in DM in 0.1 m Magnesium chloride hexahydrate, 0.1 m Tris pH 7.5, 13% w/v PEG_8000_ (C5 MemGold2), drop size 200 µL, scale bar = 100 µm (right).

All the data indicated that M363F and M363Y were more stable than the WT and P362 mutants possibly as a result of reduced conformational flexibility as reported for the UapA‐G411V. Thus, WT and both M363 mutant proteins were submitted to large scale expression and purification. It was possible to express all the proteins in large scale and successfully isolate in DDM‐based buffer. All yielded highly monodisperse size exclusion chromatography peaks (Fig. 4A,D,G) although the protein samples appeared somewhat heterogeneous on the SDS/PAGE gels (Fig. 4B,E,H). The two prominent bands indicated on the gels correspond to AtBOR1 as confirmed by mass spectrometry analysis and therefore are likely to be monomer and dimer forms of the protein. There are additional bands just below the dimer and monomer bands. These are possibly protein degradation products. Notably, these are much less prominent in the case of both M363 mutants (Fig. 4E,H) compared with the WT protein (Fig. 4B), supporting the fact that these are both more stable than the WT protein. All proteins were submitted to crystallisation trials and yielded crystals (Fig. 4C,F,I) in several conditions. Needle‐shaped crystals of AtBOR1 M363F (~ 200 µm in length) were obtained consistently in 0.1 m MOPS pH 7, 9% PEG_8000_ in a 1 µL + 1 µL drop in a sitting drop vapour diffusion set‐up. These initial crystals were harvested, cryoprotected in 30% ethylene glycol and screened at Diamond Light Source. None of the crystals had a diffraction limit less than 10 Å. Optimisation of the condition through the addition of 30% w/v D‐(+)‐glucose monohydrate yielded rod‐shaped crystals, that were more 3‐D and less fragile; typically, ~ 100µm in length (Fig. 4F Left). AtBOR1 M363F was also purified in DM, and smaller (~ 80 µm) irregular shaped crystals were obtained in A10 MemGold2 (0.2 m Li sulphate, 0.1 m Na chloride, 0.1 m HEPES pH7, 31% v/v PEG400) (Fig. 4F Right). Crystals of AtBOR1 M363Y in DDM were obtained in condition E1 MemTrans (0.2 m Ammonium acetate, 0.1 m sodium chloride, 0.1 m Bis‐Tris pH 6.3, 12% PEG_4000_) (Fig. 4I Left) and in C5 MemGold2 (0.1 m Magnesium chloride hexahydrate, 0.1 m Tris pH 7.5, 13% w/v PEG_8000_) when the mutant was purified in DM (Fig. 4I Right). Although the maximal diffraction at the moment is limited to ~ 5.5–6 Å for AtBOR1 M363F in DDM, we observed a clear improvement in the quality and diffraction limit of the crystals compared with most of our WT AtBOR1 crystals (~ 7 Å) (Fig. 4C).

## Discussion

Stabilisation by protein engineering remains one of the key tools for obtaining membrane proteins in a suitable state for structural studies. However, when working with a relatively large protein and limited resources, it can be difficult to know where to start to modify a given protein in order to produce a stable construct. In this case, we attempted to map a stabilising mutant from UapA, an SLC23 family transporter, to AtBOR1, an SLC4 family transporter. While these proteins are from different transporter families and share low overall sequence identity (16%), they do share significant structural homology as illustrated in Fig. 1. The two crystal structures available differ in conformation with UapA in the inward‐facing conformation and the AtBOR1 in the inward occluded conformation. While the two proteins superimpose overall relatively well, it was a little difficult to see which of two residues of AtBOR1 (P362 and M363) might be the direct structural equivalent of UapA G411V. Thus, we decided to mutate both sites, increasing the bulk in both cases substituting P362 to either Val or Leu and substituting M363 to Phe or Tyr.

**Fig. 5:**
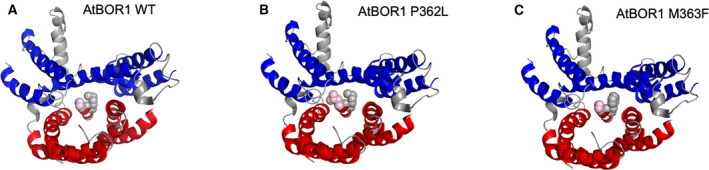
Mutations at M363 have stabilising effects. (A) WT AtBOR1 from the structure (PDB 5L25). (B) AtBOR1 P362L and (C) AtBOR1 M363F. The mutations were generated in PyMol (Schrödinger, New York, NY, USA) and the most likely position of the rotamer selected for the image. Residues at position 362 and 363 are shown in pink and grey spheres, respectively. In all structure images, only a single monomer is represented, the core domain helices are coloured red and the gate domain helices are coloured blue with interconnecting regions shown in grey. TM3 and sections of the loop connecting TM3 and TM4 have been removed for clarity.

Mutations of P362 had no major effect on the stability of the protein, suggesting that either substitutions at this site were not sufficiently bulky to block the conformational rearrangements associated with the elevator domain or that residue is in the wrong position to affect that transition. A closer look at the most likely conformation adopted by the substituted Val and Leu residues suggests that P362V or P362L do not project much further into the translocation channel than the native, adjacent M363 residue (Fig. 5). However, substitution of the M363 residue with Phe or Tyr results in a relatively longer side chain which might prevent the movement of the core domain against the gate domain (Fig. 5).

Both the M363F and M363Y proteins produce crystallisable protein which in our hands consistently results in more reproducible and slightly better diffracting crystals than the WT. It is possible that additional modifications need to be made in order to increase the resolution further. In the case of UapA, removal of a small section of the N terminus in addition to the G411V substitution was critical for successful structure determination [11, 13]. The available structure of AtBOR1 was obtained of the protein lacking the 59 C‐terminal residues. In our hands, we have found this construct to be less stable than the full‐length WT (data not shown); however, AtBOR1 does contain a large intracellular loop (~ 100 residues) between TMs 10 and 11. Removing this region or stabilising it with a nanobody might facilitate high‐resolution X‐ray crystallographic analysis. Alternatively, our conformationally restricted mutants might be suitable for cryo‐EM analysis. Our AtBOR1 proteins isolate as dimers in DDM [25], as seen in the AtBOR1 structure, unlike the ScBOR1p [23]. While the AtBOR1 dimer is still on the small side for cryo‐EM analysis, there are examples of proteins of similar molecular weight that have been successfully structurally characterised using this technique including the SLC4 protein, NBCe1 [17].

Our study indicates that it is possible to identify stabilising mutants of integral membrane transport proteins based on structurally related molecules. Mutagenesis is widely used as a means of stabilising proteins; however, it potentially involves generating and screening a large number of variants. We feel our results show that it is at the very least worth attempting to screen variants equivalent to those tried and tested in other related proteins. Importantly, in our study, we demonstrated that this approach may be successful when the proteins are very distantly related. Our findings should give researchers confidence that this approach represents one additional tool in the membrane protein researcher’s toolbox. This may facilitate structural studies of individual conformational states of transporter proteins and also provide insights into conformational dynamics using emerging techniques for membrane proteins such as hydrogen‐deuterium exchange‐mass spectrometry (HDX‐MS) [26].

## Conflict of interest

The authors declare no conflicts of interest.

## Author contributions

BB and ADC conceived the project. CC and NJS generated and characterised the mutants. TCM and ACMJ carried out some of the localisation experiments. BB wrote the manuscript with input from all other authors.

## Supporting information


**Table S1**. Oligonucleotide pair sequences used for generation of the AtBOR1 mutants.
**Fig. S1**. Expression levels, solubilisation efficiencies and localisation of WT and mutant forms of AtBOR1. The expression levels (A), localisation (B) and solubilisation efficiencies (C) of the individual AtBOR1 constructs were assessed based on the fluorescence of the fused GFP. The average expression levels ± SD of at least five independent experiments are shown (A). Representative localisation images of the individual proteins from 2 independent experiments are shown (B). In each case a clear ring of protein is evident corresponding to transporter correctly localised to the membrane. There are also highly fluorescent punctate structures visible inside the yeast cells likely to be the result of unfolded and incorrectly trafficked protein. The images were generated using a 63X Oil Objective and analysed with Fiji software. C) Solubilisation efficiencies were obtained from at least 2 independent experiments for each AtBOR1 mutant.
**Fig. S2**. Obtaining a Tm for WT AtBOR1. WT AtBOR1 was expressed as a C‐terminal GFP fusion protein in *S. cerevisiae* as described in the main manuscript, membranes were prepared and then solubilised in 1% DDM. Individual aliquots (~ 20 µg/ sample) were incubated at a range of temperatures (4, 25, 30, 35, 40, 45, 50 or 55 °C) prior to being loaded onto a Superose 6 10/300 column. The individual fractions were assessed for fluorescence indicating the presence of GFP. The peak containing monodispersed fusion protein is labelled M in panel A). The amount of monodispersed protein reduces upon heating at temperatures of 45 °C and above with an associated increase in the amount of aggregated protein (peak labelled A). The peak height was plotted against temperature (B) and this indicated that the Tm for WT AtBOR1 was ~ 43 °C.
**Fig. S3**. Functional complementation analysis of the P362V and P363L mutants. *S. cerevisiae* cells were spotted in a 5× series of dilution from left to right on media containing 0, 5, or 7 mm boric acid. Plates were incubated at 30 °C for 5 days before being imaged. FGY217 Δ*bor1*p cells were transformed with an empty vector as a negative control (FGY217 Δbor1p). FGY217 cells expressing the native ScBOR1p were used to demonstrate endogenous BOR1p activity (FGY217). FGY217 Δ*bor1p* cells overexpressing AtBOR1 WT were also analysed as a positive control.

## Data Availability

All data relevant to the manuscript are presented in the main manuscript or in the Supporting information.

## References

[1] Jardetzky O (1966) Simple allosteric model for membrane pumps. Nature 211, 969–970. 5968307 10.1038/211969a0

[2] Drew D and Boudker O (2016) Shared molecular mechanisms of membrane transporters. Annu Rev Biochem 85, 543–572. 27023848 10.1146/annurev-biochem-060815-014520

[3] Abramson J , Smirnova I , Kasho V , Verner G , Kaback HR and Iwata S (2003) Structure and mechanism of the lactose permease of *Escherichia coli* . Science 301, 610–615. 12893935 10.1126/science.1088196

[4] Errasti‐Murugarren E , Fort J , Bartoccioni P , Díaz L , Pardon E , Carpena X , Espino‐Guarch M , Zorzano A , Ziegler C , Steyaert J *et al*. (2019) L amino acid transporter structure and molecular bases for the asymmetry of substrate interaction. Nat Commun 10, 1807–1812. 31000719 10.1038/s41467-019-09837-z PMC6472337

[5] Coleman JA , Green EM and Gouaux E (2016) X‐ray structures and mechanism of the human serotonin transporter. Nature 532, 334–339. 27049939 10.1038/nature17629 PMC4898786

[6] Coleman JA and Gouaux E (2018) Structural basis for recognition of diverse antidepressants by the human serotonin transporter. Nat Struct Mol Biol 25, 170–175. 29379174 10.1038/s41594-018-0026-8 PMC5962350

[7] Penmatsa A , Wang KH and Gouaux E (2013) X‐ray structure of dopamine transporter elucidates antidepressant mechanism. Nature 503, 85–90. 24037379 10.1038/nature12533 PMC3904663

[8] Garaeva AA , Oostergetel GT , Gati C , Guskov A , Paulino C and Slotboom DJ (2018) Cryo‐EM structure of the human neutral amino acid transporter ASCT2. Nat Struct Mol Biol 25, 515–521. 29872227 10.1038/s41594-018-0076-y

[9] Garaeva AA , Guskov A , Slotboom DJ and Paulino C (2019) A one‐gate elevator mechanism for the human neutral amino acid transporter ASCT2. Nat Commun 10, 1–8. 31366933 10.1038/s41467-019-11363-x PMC6668440

[10] Canul‐Tec JC , Assal R , Cirri E , Legrand P , Brier S , Chamot‐Rooke J and Reyes N (2017) Structure and allosteric inhibition of excitatory amino acid transporter 1. Nature 544, 446–451. 28424515 10.1038/nature22064 PMC5410168

[11] Alguel Y , Amillis S , Leung J , Lambrinidis G , Capaldi S , Scull NJ , Craven G , Iwata S , Armstrong A , Mikros E *et al*. (2016) Structure of eukaryotic purine/H(+) symporter UapA suggests a role for homodimerization in transport activity. Nat Commun 7, 11336–11339. 27088252 10.1038/ncomms11336 PMC4837479

[12] Leung J , Karachaliou M , Alves C , Diallinas G and Byrne B (2010) Expression and purification of a functional uric acid‐xanthine transporter (UapA). Protein Expr Purif 72, 139–146. 20153431 10.1016/j.pep.2010.02.002

[13] Leung J , Cameron AD , Diallinas G and Byrne B (2013) Stabilizing the heterologously expressed uric acid‐xanthine transporter UapA from the lower eukaryote *Aspergillus nidulans* . Mol Membr Biol 30, 32–42. 22694048 10.3109/09687688.2012.690572

[14] Koukaki M , Vlanti A , Goudela S , Pantazopoulou A , Gioule H , Tournaviti S and Diallinas G (2005) The nucleobase‐ascorbate transporter (NAT) signature motif in UapA defines the function of the purine translocation pathway. J Mol Biol 350, 499–513. 15953615 10.1016/j.jmb.2005.04.076

[15] Papageorgiou I , Gournas C , Vlanti A , Amillis S , Pantazopoulou A and Diallinas G (2008) Specific interdomain synergy in the UapA transporter determines its unique specificity for uric acid among NAT carriers. J Mol Biol 382, 1121–1135. 18718842 10.1016/j.jmb.2008.08.005

[16] Arakawa T , Kobayashi‐Yurugi T , Alguel Y , Iwanari H , Hatae H , Iwata M , Abe Y , Hino T , Ikeda‐Suno C , Kuma H *et al*. (2015) Crystal structure of the anion exchanger domain of human erythrocyte band 3. Science 350, 680–684. 26542571 10.1126/science.aaa4335

[17] Huynh KW , Jiang J , Abuladze N , Tsirulnikov K , Kao L , Shao X , Newman D , Azimov R , Pushkin A , Zhou ZH *et al*. (2018) CryoEM structure of the human SLC4A4 sodium‐coupled acid‐base transporter NBCe1. Nat Commun 9, 900. 29500354 10.1038/s41467-018-03271-3 PMC5834491

[18] Thurtle‐Schmidt BH and Stroud RM (2016) Structure of Bor1 supports an elevator transport mechanism for SLC4 anion exchangers. Proc Natl Acad Sci USA 113, 10542–10546. 27601653 10.1073/pnas.1612603113 PMC5035872

[19] Wang C , Sun B , Zhang X , Huang X , Zhang M , Guo H , Chen X , Huang F , Chen T , Mi H *et al*. (2019) Structural mechanism of the active bicarbonate transporter from cyanobacteria. Nat Plants 5, 1184–1193. 31712753 10.1038/s41477-019-0538-1

[20] Geertsma ER , Chang Y‐N , Shaik FR , Neldner Y , Pardon E , Steyaert J and Dutzler R (2015) Structure of a prokaryotic fumarate transporter reveals the architecture of the SLCfamily. Nature Struc Mol Biol 22, 803–808. 10.1038/nsmb.3091 26367249

[21] Drew D , Newstead S , Sonoda Y , Kim H , von Heijne G and Iwata S (2008) GFP‐based optimization scheme for the overexpression and purification of eukaryotic membrane proteins in *Saccharomyces cerevisiae* . Nat Protoc 3, 784–798. 18451787 10.1038/nprot.2008.44 PMC2744353

[22] Saouros S , Cecchetti C , Jones A , Cameron AD and Byrne B (2020) Strategies for successful isolation of a eukaryotic transporter. Protein Expr Purif 166, 105522. 31654736 10.1016/j.pep.2019.105522

[23] Pyle E , Guo C , Hofmann T , Schmidt C , Ribiero O , Politis A and Byrne B (2019) Protein‐lipid interactions stabilize the oligomeric state of BOR1p from *Saccharomyces cerevisiae* . Anal Chem 91, 13071–13079. 31513392 10.1021/acs.analchem.9b03271

[24] Pettersen EF , Goddard TD , Huang CC , Couch GS , Greenblatt DM , Meng EC and Ferrin TE (2004) UCSF Chimera–a visualization system for exploratory research and analysis. J Comput Chem 25, 1605–1612. 15264254 10.1002/jcc.20084

[25] Bae HE , Cecchetti C , Du Y , Katsube S , Mortensen JS , Huang W , Rehan S , Lee HJ , Loland CJ , Guan L *et al*. (2020) Pendant‐bearing glucose‐neopentyl glycol (P‐GNG) amphiphiles for membrane protein manipulation: Importance of detergent pendant chain for protein stabilization. Acta Biomater 112, 250–261. 32522715 10.1016/j.actbio.2020.06.001 PMC7366829

[26] Martens C , Shekhar M , Borysik AJ , Lau AM , Reading E , Tajkhorshid E , Booth PJ and Politis A (2018) Direct protein‐lipid interactions shape the conformational landscape of secondary transporters. Nat Commun 9, 4151. 30297844 10.1038/s41467-018-06704-1 PMC6175955

