# Supplementary material for: Transfer of stabilising mutations between different secondary active transporter families
Source: FEBS Open Bio. 2021 May 8;11(6):1685–94. doi: 10.1002/2211-5463.13168 (PMC8167854; doi:10.1002/2211-5463.13168)
Supplement: Supplementary file 1 — Table S1. Oligonucleotide pair sequences used for generation of the AtBOR1 mutants. Fig. S1. Expression levels, solubilisation efficiencies and localisation of WT and mutant forms of AtBOR1. The expression levels (A), localisation (B) and solubilisation efficiencies (C) of the individual AtBOR1 constructs were assessed based on the fluorescence of the fused GFP. The average expression levels ± SD of at least five independent experiments are shown (A). Representative localisation images of the individual proteins from 2 independent experiments are shown (B). In each case a clear ring of protein is evident corresponding to transporter correctly localised to the membrane. There are also highly fluorescent punctate structures visible inside the yeast cells likely to be the result of unfolded and incorrectly trafficked protein. The images were generated using a 63X Oil Objective and analysed with Fiji software. C) Solubilisation efficiencies were obtained from at least 2 independent experiments for each AtBOR1 mutant. Fig. S2. Obtaining a Tm for WT AtBOR1. WT AtBOR1 was expressed as a C‐terminal GFP fusion protein in S. cerevisiae as described in the main manuscript, membranes were prepared and then solubilised in 1% DDM. Individual aliquots (~ 20 µg/ sample) were incubated at a range of temperatures (4, 25, 30, 35, 40, 45, 50 or 55 °C) prior to being loaded onto a Superose 6 10/300 column. The individual fractions were assessed for fluorescence indicating the presence of GFP. The peak containing monodispersed fusion protein is labelled M in panel A). The amount of monodispersed protein reduces upon heating at temperatures of 45 °C and above with an associated increase in the amount of aggregated protein (peak labelled A). The peak height was plotted against temperature (B) and this indicated that the Tm for WT AtBOR1 was ~ 43 °C. Fig. S3. Functional complementation analysis of the P362V and P363L mutants. S. cerevisiae cells were spotted in a 5× series of dilution from [file FEB4-11-1685-s001.docx]

**Supplementary Information**

**Cecchetti et al, Transfer of stabilising mutations between different secondary active transporter families**

**Table 1** Oligonucleotide pair sequences used for generation of the AtBOR1 mutants

| **Mutation** | **Oligonucleotide primer pairs (5’-3’)** |
| --- | --- |
| P362V | F: cggtgtcattcctcaatctGTAatgcataccaagagcttagc  R: gctaagctcttggtatgcatTACagattgaggaatgacaccg |
| P362L | F: cggtgtcattcctcaatctTTAatgcataccaagagcttagc  R: gctaagctcttggtatgcatTAAagattgaggaatgacaccg |
| M363F | F: gtgtcattcctcaatctccaTTTcataccaagagcttagcaac  R: gttgctaagctcttggtatgAAAtggagattgaggaatgacac |
| M363Y | F: ggtgtcattcctcaatctccaTACcataccaagagcttagcaact  R: agttgctaagctcttggtatgGTAtggagattgaggaatgacacc |

Forward (F) and reverse (R) primers are shown with the altered nucleotides indicated in uppercase.

**Supplementary Figure 1** Expression levels, solubilisation efficiencies and localisation of WT and mutant forms of AtBOR1.


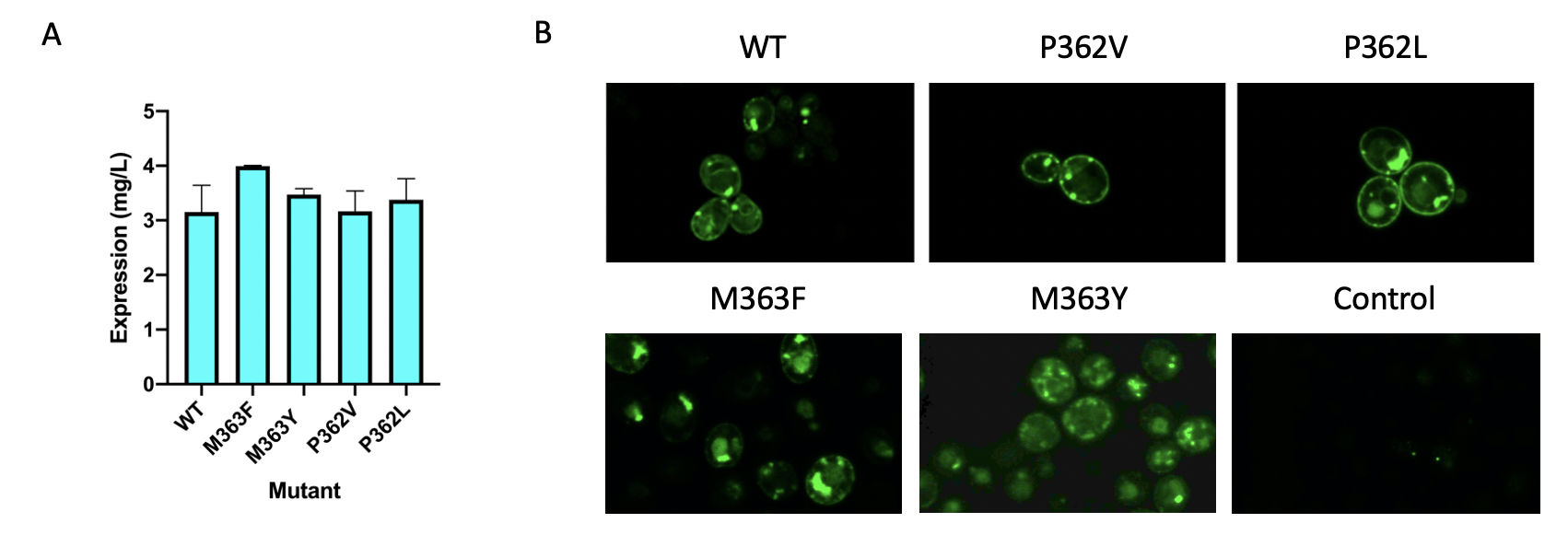


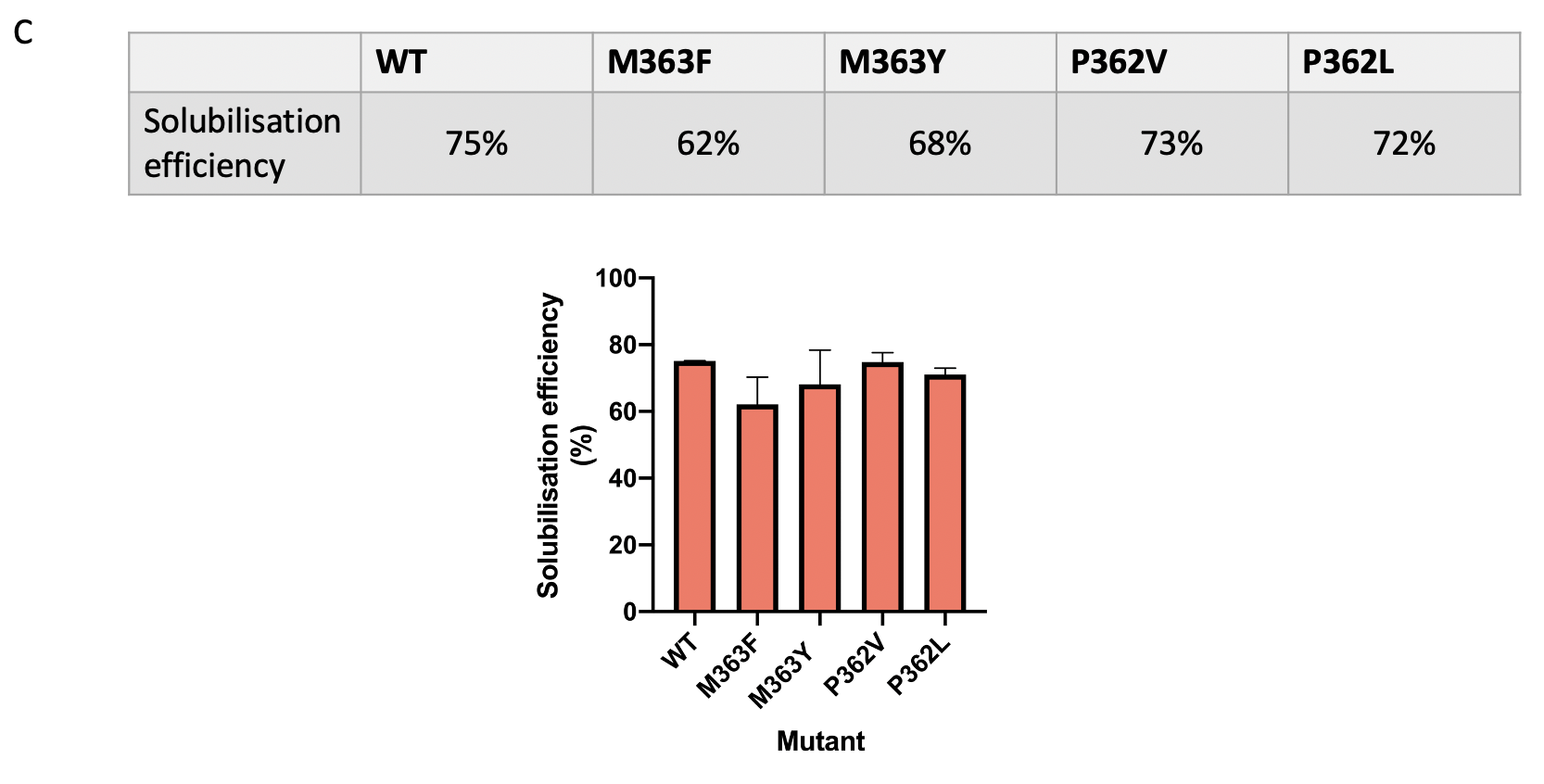


The expression levels (A), localisation (B) and solubilisation efficiencies (C) of the individual AtBOR1 constructs were assessed based on the fluorescence of the fused GFP. The average expression levels +/- S.D. of at least 5 independent experiments are shown (A). Representative localisation images of the individual proteins from 2 independent experiments are shown (B). In each case a clear ring of protein is evident corresponding to transporter correctly localised to the membrane. There are also highly fluorescent punctate structures visible inside the yeast cells likely to be the result of unfolded and incorrectly trafficked protein. The images were generated using a 63X Oil Objective and analysed with Fiji software. C) Solubilisation efficiencies were obtained from at least 2 independent experiments for each AtBOR1 mutant.

**Supplementary Figure 2** Obtaining a Tm for WT AtBOR1

**
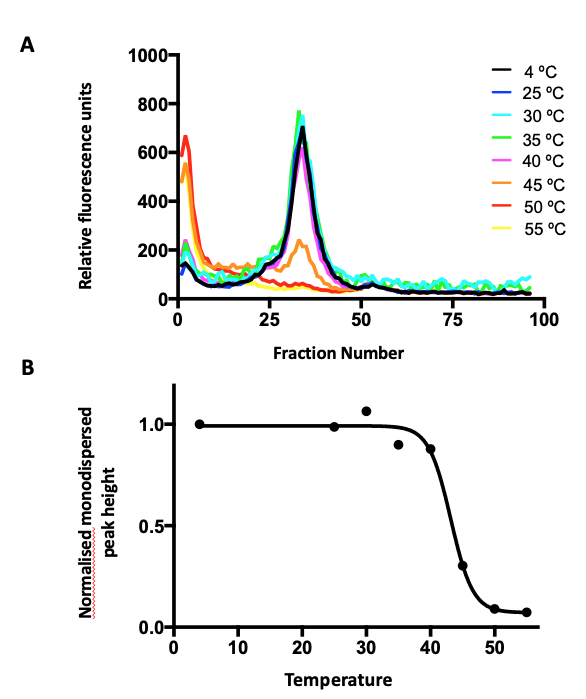
**

WT AtBOR1 was expressed as a C-terminal GFP fusion protein in *S. cerevisiae* as described in the main manuscript, membranes were prepared and then solubilised in 1% DDM. Individual aliquots (~20 µg/ sample) were incubated at a range of temperatures (4, 25, 30, 35, 40, 45, 50 or 55 ºC) prior to being loaded onto a Superose 6 10/300 column. The individual fractions were assessed for fluorescence indicating the presence of GFP. The peak containing monodispersed fusion protein is labelled M in panel A). The amount of monodispersed protein reduces upon heating at temperatures of 45 ºC and above with an associated increase in the amount of aggregated protein (peak labelled A). The peak height was plotted against temperature (B) and this indicated that the Tm for WT AtBOR1 was ~43 ºC.

**Supplementary Figure 3** Functional complementation analysis of the P362V and P363L mutants.


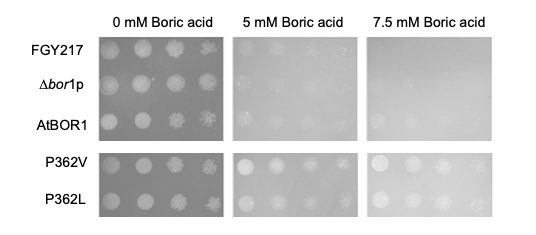


*S. cerevisiae* cells were spotted in a 5× series of dilution from left to right on media containing 0, 5, or 7 mM boric acid. Plates were incubated at 30 °C for 5 days before being imaged. FGY217 Δ*bor1*p cells were transformed with an empty vector as a negative control (FGY217 Δbor1p). FGY217 cells expressing the native ScBOR1p were used to demonstrate endogenous BOR1p activity (FGY217). FGY217 Δ*bor1p* cells overexpressing AtBOR1 WT were also analysed as a positive control.
